# Datasets on abundance of common blossom thrips and weather variables in small-scale avocado orchards at Taita Hills and Mount Kilimanjaro

**DOI:** 10.1016/j.dib.2017.09.051

**Published:** 2017-09-27

**Authors:** James J. Odanga, Samira Mohamed, Florence Olubayo, Richard Nyankanga, Sizah Mwalusepo, Sevgan Subramanian, Tino Johansson, Sunday Ekesi

**Affiliations:** a ICIPE-African Insect Science for Food and Health, P.O. Box 30772-00100, Nairobi, Kenya; b Department of Plant Science and Crop Protection, University of Nairobi, P.O. Box 30197-00100, Nairobi, Kenya; c Invertebrate Zoology Section, National Museums of Kenya, P.O. Box 40658-00100, Nairobi, Kenya; d Department of General Studies, Dar es Salaam Institute of Technology, P.O. Box 2958, Dar es Salaam, Tanzania; e Department of Geosciences and Geography, University of Helsinki, P.O. Box 68, FI-00014, Finland

**Keywords:** *Frankliniella schultzei*, Avocado, Weather variables, Taita Hills, Mount Kilimanjaro

## Abstract

Avocado, *Persea americana* Miller (Lauraceae), is an important fruit crop cultivated by small-holder farmers along Afrotropical highlands of Taita Hills in South-eastern Kenya and Mount Kilimanjaro in Northern Tanzania. The small-holder farmers in these East African regions generate substantial food and cash from avocado fruits. However, the avocado crop is faced with challenges of infestation by insect pests such as the common blossom thrips (*Frankliniella schultzei* Trybom) which feeds on pollen and floral tissue thereby reducing productivity of the trees. Moreover, there is no information describing distribution patterns of *Frankliniella schultzei* and associated weather in East African avocado orchards despite the fact that small-scale farming is dependent on rainfall. This article was, therefore, initiated to provide dataset on abundance of *Frankliniella schultzei* from the avocado plants that relates with monthly rainfall and air temperatures at Taita Hills and Mount Kilimanjaro. *Frankliniella schultzei* was collected using white coloured beating tray and camel brush whereas air temperatures (°C) and rainfall (mm) was recorded daily using automatic data loggers and rain gauge, respectively. The survey at the two transects commenced during peak flowering season of avocado crop in August up to end of harvesting period in July of the following year. Temporal datasets were generated by Kruskal-Wallis Chi-square test. Current temporal datasets presents strong baseline information specifically for Kenya and Tanzania government agencies to develop further agricultural strategies aimed at improving avocado farming within Taita Hills and Mount Kilimanjaro agro-ecosystems.

**Specifications Table**

**Table:** 

Subject area	Horticulture and weather variability
More specific subject area	Temporal dataset
Type of data	Figures
How data was acquired	Field survey was carried out in small-scale avocado farmlands; air temperature (°C) was recorded daily using data loggers whereas rainfall was recorded by using rain gauge at Taita Hills and Mount Kilimanjaro.
Data format	Analyzed
Experimental factors	Abundance of *Frankliniella schultzei* was sampled monthly from avocado trees along the study areas.
Experimental features	Temporal datasets were generated by Kruskal-Wallis Chi-square test.
Data source location	Taita Hills in Kenya and Mount Kilimanjaro in Tanzania
Data accessibility	Data are available in this article

**Value of the data**

• Abundance datasets of *Frankliniella schultzei* presents baseline information specifically for Kenya and Tanzania government agencies, scientists and avocado farmers to enhance agricultural strategies.
• Weather datasets contributes to precise climate information of Taita Hills and Mount Kilimanjaro.
• The weather variables may be used to relate with avocado plant phenology (flowering, fruiting and harvesting) and also seasonal abundance of pest or beneficial insect species.

## 1 Data

**Fig. 1:**
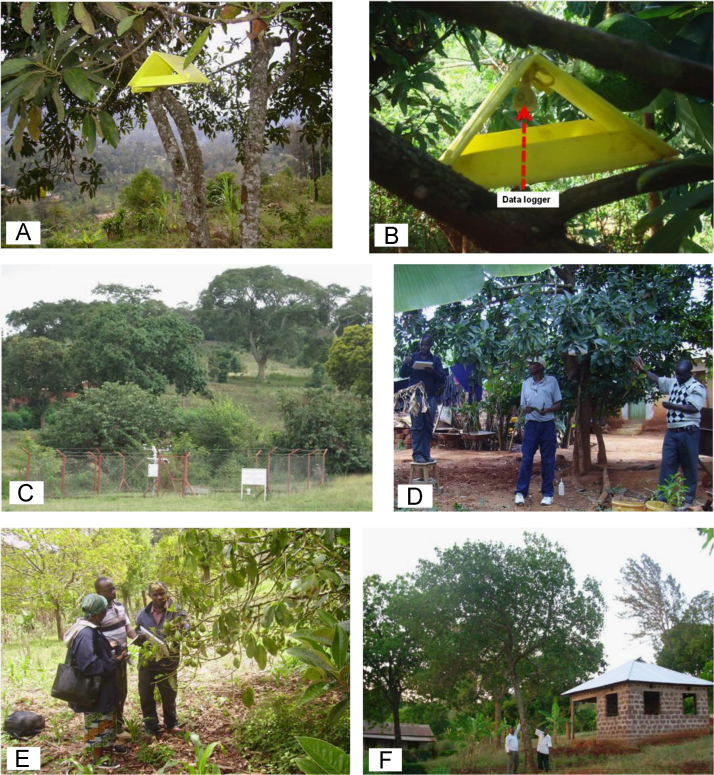
Data collection in Taita Hills and Mount Kilimanjaro areas; (A) and (B) shows data loggers, (C) Rain gauge, (D) and (E) sampling thrips and observing avocado plant phenology and (F) small-holder avocado farmland.

**Fig. 2:**
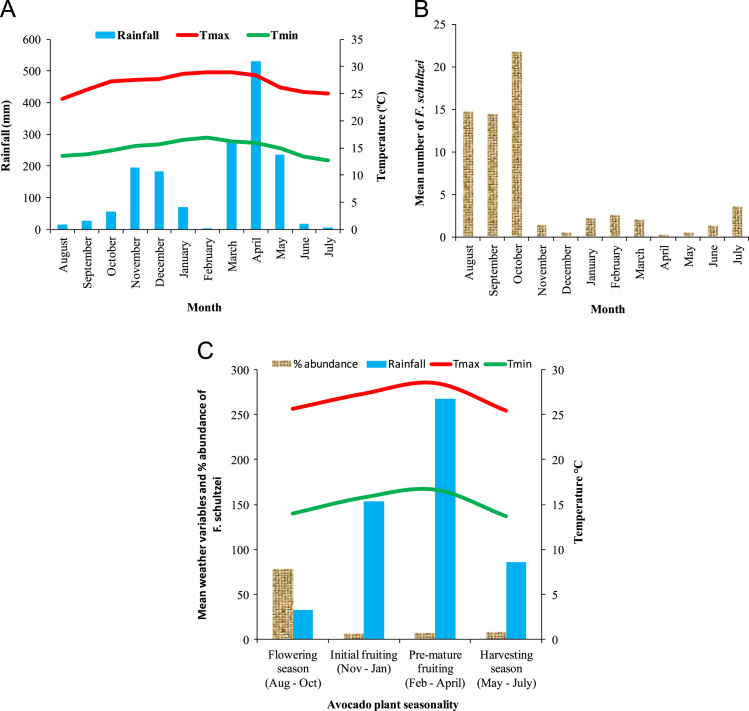
Temporal variation of *Frankliniella schultzei* and weather variables at Taita Hills in Kenya; (A) Variation of rainfall, monthly maximum (Tmax) and minimum (Tmin) temperatures for twelve months from August to July of the following year in avocado farmlands; (B) Mean abundance of *Frankliniella schultzei* in avocado orchards; (C) seasonal variation of mean abundance of *Frankliniella schultzei*, rainfall, maximum temperature (Tmax) and minimum temperature (Tmin) within avocado plant phenological events.

**Fig. 3:**
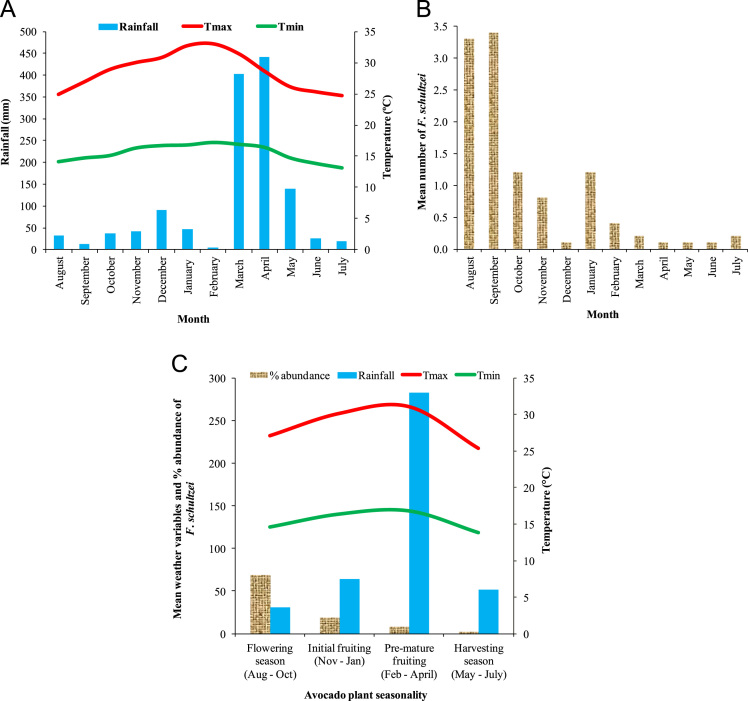
Temporal variation of *Frankliniella schultzei*.and weather variables at Mount Kilimanjaro in Tanzania; (A) Variation of rainfall, monthly maximum (Tmax) and minimum (Tmin) temperatures for twelve months from August to July of the following year in avocado farmlands; (B) Mean abundance of *Frankliniella schultzei* in avocado orchards; (C) seasonal variation of mean abundance of *Frankliniella schultzei*, rainfall, maximum temperature (Tmax) and minimum temperature (Tmin) within avocado plant phenological events.

**Fig. 4:**
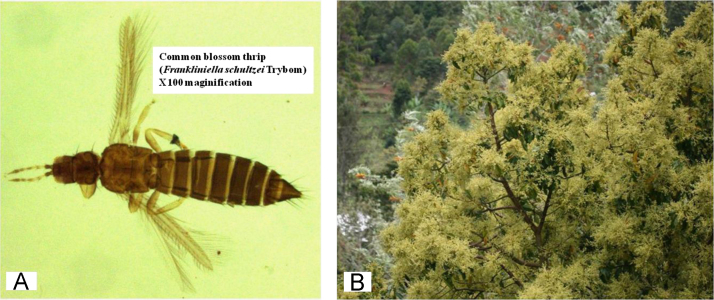
(A) The image of common blossom thrips (*Frankliniella schultzei* Trybom), and (B) a blossomed avocado host plant of the thrips at Taita Hills.

Fig. 1A and B demonstrate height and strategic location of data loggers used for recording temperature on avocado tree. Fig. 1C shows rain gauge where rainfall data was sampled. Fig. 1D and E show assessment of avocado plant phenology and sampling of thrips. Fig. 1F show small-holder avocado farmland at lowlands of the Mount Kilimanjaro transect in Tanzania. Figs. 2A and 3A shows variation of rainfall, monthly maximum and minimum temperatures for twelve months from August to July of the following year in avocado farmlands of Taita Hills and Mount Kilimanjaro, respectively. Figs. 2B and 3B indicates mean abundance of *Frankliniella schultzei* in avocado orchards at Taita Hills and Mount Kilimanjaro, respectively. Figs. 2C and 3C shows seasonal variation of mean abundance of *Frankliniella schultzei*, rainfall, maximum temperature (Tmax), and minimum temperature (Tmin) in avocado orchards at Taita Hills and Mount Kilimanjaro, respectively. Fig. 4A indicate image of common blossom thrip (*Frankliniella schultzei* Trybom) specimen and Fig. 4B show blossomed avocado plant at Taita Hills.

## 2 Experimental design, material and methods

This study was carried out in small-scale avocado farmlands at Taita Hills in South-eastern Kenya and at the South-eastern slopes of Mount Kilimanjaro in Northern Tanzania as described by [1]. The two study areas namely; Taita Hills and Mount Kilimanjaro are situated 90 km apart and both are about 150 km from Indian Ocean. Avocado plant is the most important fruit crop cultivated by small-holder farmers in the study areas along altitudinal gradient from 900 to 1800 m above sea level.

Air temperature (°C) of avocado orchards was recorded daily along the study areas using data loggers. The data loggers were hang on the lower canopy of avocado trees at a height of 2 m. Rainfall records was obtained from rain gauge for two years between August 2012 and July 2014. Geographical coordinates and elevation of study areas was verified using Geographical position system (GPS) Garmin model eTrex 30. The common blossom thrips, *Frankliniella schultzei* Trybom, was sampled monthly from avocado trees using a white coloured beating tray and camel brush for two years as described by Palmer [2]. Mounted thrips specimens were identified at the National Museums of Kenya entomology laboratory in Nairobi using taxonomic manuals [3], [4]. The fully identified and confirmed thrip species were deposited in the entomology collection at the National Museums of Kenya. Seasonal weather patterns and abundance of thrips were averaged from monthly datasets.
